# Ultrasonography is a reliable clinical method for assessing biological maturation

**DOI:** 10.1186/s13102-026-01649-1

**Published:** 2026-03-17

**Authors:** Claes Göran Sundell, Apostolos Theos, Ann-Sofie Lindberg, Anna-Clara Rullander, Elena Lundberg

**Affiliations:** 1 Department of Community Medicine and Rehabilitation, Sports medicine, Umeå University, Umeå, Sweden; 2 Department of Health, Education and Technology, Lulea University of Technology, Luleå, Sweden; 3 Department of Nursing, Umeå University, Umeå, Sweden; 4 Department of Clinical Sciences, Pediatrics, Umeå University, Umeå, Sweden; 5 Umeå School of Sport Sciences, Umeå University, Umeå, Sweden

**Keywords:** Tibial Tuberosity, Child, Adolescent, Sports Medicine, Growth and Maturation

## Abstract

**Background:**

Accurate assessment of biological maturity (BM) is essential in pediatric sports medicine to support individualized training and injury prevention. Traditional methods, such as sexual maturity ratings (SMR) and radiographic imaging, have limitations including subjectivity, discomfort, and radiation exposure. This study aimed to evaluate and validate the use of two established ultrasonography protocols for assessing biological maturity at the tibial tuberosity in healthy, physically active youth.

**Methods:**

Seventy-three children and adolescents (41 boys, 32 girls; mean age 13.4 years) were recruited from schools in Northern Sweden. BM was assessed using SMR and two established ultrasonographic protocols (Sailly and Kijima). Validity was evaluated using Spearman’s rho, and inter- and intrarater reliability were assessed with Cohen’s weighted kappa.

**Results:**

Ultrasonographic assessments showed strong agreement with SMR-based evaluations. Interrater reliability was substantial for both methods (κ = 0.761–0.814, *p* < 0.001). The method proved feasible for use by trained physiotherapists and did not require radiological or endocrinological expertise.

**Conclusion:**

Ultrasonography, combined with anthropometric data, is a valid, reliable, and practical alternative for assessing BM in youth. Its non-invasive, radiation-free nature and scalability make it particularly suitable for pediatric and sports settings. Further research should explore its application in diverse populations and its role in guiding biologically informed training strategies.

## Introduction

Biological maturation (BM) is a genetically and hormonally regulated process that facilitates the physical transition from childhood to adulthood, playing a critical role in the development of secondary sexual characteristics and overall growth. In contrast to chronological age (CA), which is determined solely by an individual’s date of birth, BM encompasses physiological development, including skeletal and pubertal stages.

Given the substantial variation in biological maturation among individuals of the same chronological age, maturation status offers a more precise criterion for categorizing youth athletes. This approach fosters healthier developmental pathways and helps mitigate the risk of injury.

Biological maturation is a dynamic process that persists throughout growth, particularly during puberty, a critical period during which the majority of skeletal mass is attained [1–3]. This process aligns more closely with key pubertal milestones, such as growth spurts and the onset of menarche, than with CA, and is influenced by a multitude of factors, including sex, nutrition, genetics, and overall health. It is observed that girls generally mature earlier than boys, a phenomenon attributable to inherent physiological differences that emerge during puberty [3–6]. Various assessment methodologies have been employed to evaluate BM, including skeletal age determination via radiographic imaging, dental age assessment, and the Sexual Maturity Rating (SMR), which classifies individuals as delayed, average, or advanced in comparison to their CA [7–9].

The Sexual Maturity Rating (SMR), commonly known as Tanner staging, is extensively utilized for the assessment of sexual maturation. However, despite its widespread application, this method is inherently subjective, may induce discomfort, and 

exhibits variability in reliability across different populations. More accurate evaluations often necessitate supplementary techniques, such as longitudinal growth monitoring [6, 10–12] and radiographic assessments, including wrist X-rays [9, 13]. These techniques, however, require expert interpretation and expose individuals to radiation, which restricts their feasibility in large-scale youth sports settings.

As a non-invasive alternative to radiographic methods, ultrasound imaging has gained prominence as a tool for assessing skeletal maturity, particularly through the examination of the tibial tuberosity during various developmental stages [14, 15]. Ultrasound proves especially beneficial in monitoring development and evaluating injury risk among young athletes [16].

In the context of sports, the prevailing practice of categorizing athletes by age predominantly utilizes CA, which frequently does not provide an accurate representation of the athletes’ developmental status. This discrepancy is particularly pronounced among female athletes through the ages of 9 to- 13, as well as male athletes between the ages of 11 and 15 [14, 15]. Such athletes are often more vulnerable to skeletal injuries, although they may demonstrate greater long-term athletic potential [17]. The role of BM as a significant biomarker for assessing growth plate and pubertal maturity is gaining recognition within the disciplines of youth sports medicine and training [18–20]. As a result, there is a growing demand for practical, non-radiological methods to evaluate biological maturity that do not require specialized expertise in radiology or endocrinology. Such methodologies are particularly essential for managing large cohorts within youth sports settings.

## Aim

This study aimed to evaluate and validate the use of two established ultrasonography protocols for assessing BM at the tibial tuberosity in healthy, physically active youth. Specifically, we examined the validity and reliability of an ultrasonography -based protocol for visualizing the stages of BM at the tibial tuberosity.

We hypothesized that the ultrasonography protocol could be executed reliably by two independent investigators and that the results would exhibit a significant correlation with Sexual Maturity Rating.

## Materials and methods

### Participants

Seventy-three healthy, physically active children and adolescents (engaging in more than two hours of activity per week) were enrolled between November 2023 and April 2024. Participants were recruited via convenience sampling from municipal primary schools in Umeå and Skellefteå, where physical education teachers and school nurses provided study information to eligible students and their parents or guardians.

Inclusion criteria were: [1] healthy children and adolescents, and [2] enrolled in participating municipal primary schools in Northern Sweden.Exclusion criteria were: [1] current or previous injuries affecting the tibial tuberosity, and [2] any musculoskeletal condition that could influence knee function or training participation.

Written informed consent was obtained from the children and the legal guardians/parents after information about the study. The study was approved by the Swedish Ethical Review Authority (EPM DNR: Dnr 2023-00341-01) and was conducted in accordance with the Declaration of Helsinki.

### General design

The entitled participants were evaluated by a pediatric endocrinologist and by a physiotherapist at the same visit, independently. The pediatric endocrinologist evaluated participants in accordance with SMR [7, 8]. Subsequently, the physiotherapist performed ultrasound examination of the tibial tuberosity according to Sailly et al. and Kijima et al. [14, 15]. Sexual maturity rating was chosen as the reference standard because this study aimed to assess non-invasive, practical methods for determining biological maturation in youth sports. While wrist X-ray is the gold standard for skeletal age, it was excluded due to ethical, logistical, and feasibility concerns in school and club settings. Sexual maturity rating is commonly used in pediatric and sports medicine, aligns well with radiographic bone age, and is suitable for this context. The main contribution of this study is the direct comparison of two established staging systems and their relationship to SMR defined maturity within a single group.

### Measurements

#### Growth and maturation

Anthropometric data about height and weight in participants were followed previously at school healthcare units. Both measurements were conducted at the same time and ahead of the SMR and ultrasound evaluations. Body height was measured with a validated stadiometer (Seca 878, Seca GmbH & Co, Hamburg, Germany. accurate to 0.1 cm), the mean of three independent measurements with traction of standing height was obtained, mainly by the same nurse/doctor. Body weight was measured with an electronic scale (Seca 878, ISO 9001 and ISO 13485 with precision of 0.1 kg) wearing light underwear. Puberty was self-assessed by the adolescent with assistance of pediatric endocrinologist, according to Tanner scale (breast development for the girls and debut of menarche, genitalia development for the boys) [7, 8] and testicular size according to Prader [21], SMR was estimated/assessed after summary of above mentioned factors.

#### Ultrasound examination

**Fig. 1:**
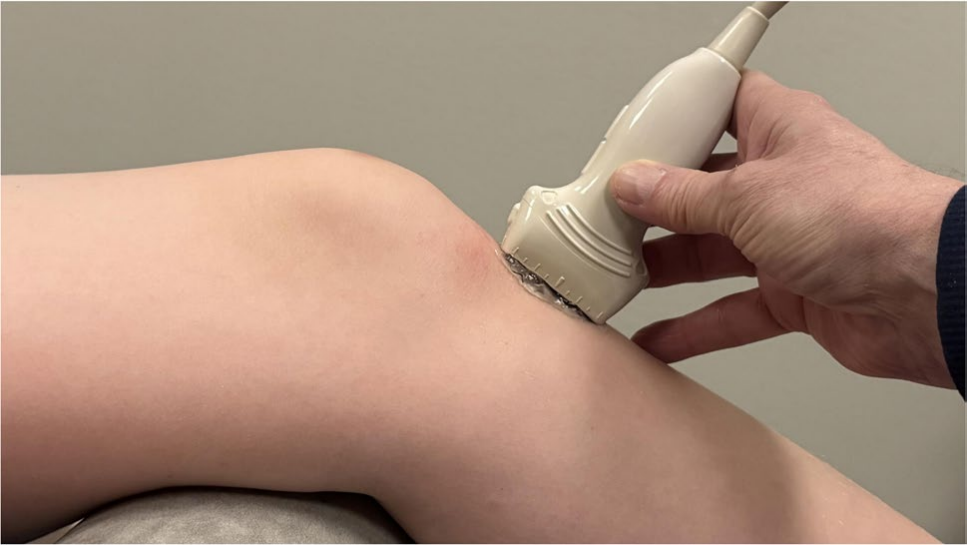
Ultrasound examination of the tibial tuberosity using a linear-array transducer

An ultrasonography machine (Hitachi ALOKA Noblus Ultrasound Diagnostic Scanner, Japan) was used together with a linear probe (12–18 MHz). Participants were lying supine on a bench with 40 degrees flexed knees (Fig. 1).

**Fig. 2:**
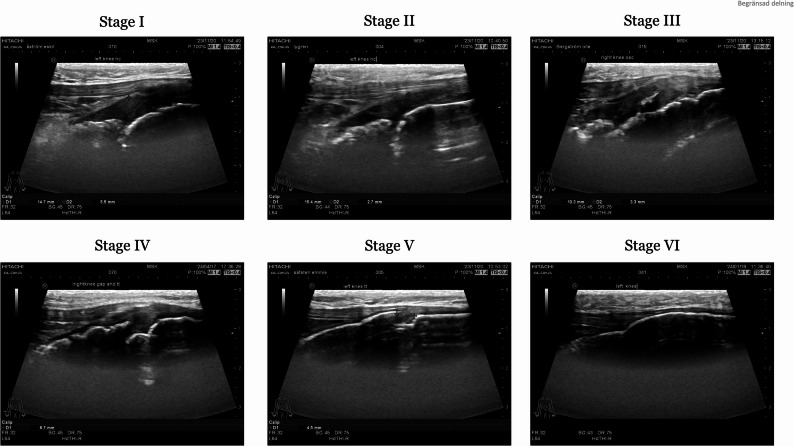
Ultrasonographic appearance of the tibial tuberosity across developmental stages as described by Kijima et al. Sequential images illustrate Stage I through Stage VI, demonstrating progressive morphological changes in the ossification center

The longitudinal mode was performed with the probe placed over the tibial tuberosity with the probe mark heading proximally and a scan from the medial to the lateral part of the tibial tuberosity. The ultrasound examination was performed on both legs starting with the left tibial tuberosity and followed by the right-side tibial tuberosity. Ultrasonography scan was used according to previously described protocol [14, 15] to assess the stage of skeletal maturation in the tibial tuberosity (Fig. 2). The original ultrasound protocols established by Sailly et al. and Kijima et al. were strictly adhered to, with each methodology executed as outlined in their respective publications. For the protocol described by Sailly et al., assessment was conducted using the following staging: Stage 1—cartilaginous tuberosity; Stage 2—emergence of a fragmented or irregular secondary ossification center, potentially with Doppler positivity; Stage 3—progressive fusion of the ossification center; and Stage 4—complete bony fusion. In accordance with Kijima et al., evaluation involved: Stage 1—pre-ossification; Stage 2—early ossification; Stage 3—progressive ossification/enlargement; Stage 4—apophyseal stage; Stage 5—epiphyseal stage; and Stage 6—complete fusion. The tibial tuberosity was chosen for assessment because its superficial apophyseal structure allows for predictable maturation during adolescence, making it easy to observe and especially suitable for non-invasive maturity screening among young athletes. To evaluate the reliability of ultrasonographic assessments, both inter-rater and intra-rater reliability analyses were conducted. Specifically, two clinicians, each with over ten years of experience in pediatric knee ultrasound, independently evaluated anonymized images according to the standardized criteria of the Sailly and Kijima protocols. Prior to assessment, both raters participated in a calibration session to ensure interpretative consistency. All evaluations were conducted in a blinded and randomized manner.

### Analysis and statistics

No formal a priori power calculation was performed due to the exploratory nature and school-based feasibility constraints. With *n* = 73, the 95% CI half-width for a standardized mean is ~ 0.23 SD, and the detectable standardized effects (α = 0.05, 1 − β = 0.80) are d ≈ 0.33 for one-sample/paired and d ≈ 0.46 for two-group comparisons; for correlations, *r* ≈ 0.30–0.35 is detectable. These benchmarks indicate adequate precision and power for moderate effects.

Descriptive statistics were used to summarize the characteristics of the study population. Validity was assessed by examining correlations between ordinal variables using Spearman’s rho (ρ) with 95% confidence intervals, focusing on the agreement between SMR and two ultrasonographic methods [14, 15].

Inter- and intra-rater reliability of the ultrasound assessments were evaluated using Cohen’s weighted kappa [22] with 95% confidence intervals. Inter-rater reliability reflected the consistency between two independent, blinded investigators. Intra-rater reliability was determined by blinded reevaluation of 50 randomly selected images by the same investigator.

Statistical significance was set at α = 0.05. All analyses were conducted using SPSS v.29 (IBM, Armonk, NY, USA).

## Results

### Participants

The study included 73 participants: 41 boys (56%) and 32 girls (44%), with a mean age of 13.4 years (range 10.4–16.6). Boys had a mean age of 14.0 years, and girls 12.8 years. Twenty-four participants (32.9%) were prepubertal or in early puberty, evenly distributed between sexes. The remaining 49 participants (67.1%) were in later puberty, comprising 29 boys and 20 girls (Table 1).

**Table 1:** Characteristics of the study group

	*N* (% of total)	Age (years) Mean (min-max)	Body Weight (kg) Mean (min-max)	Body Height (cm) Mean (min-max)	SMR Stages: I–II	SMR Stages: III–IV
Females	32 (44%)	12.8 (10.5–16.0)	51.0 (30.7–67.1)	160.2 (138.0–176.5)	12	20
**Males**	41 (56%	14.0 (10.4–16.6)	55.1 (34.5–93.8)	167.7 (146.2–187.0)	12	29
**Total**	73 (100%)	13.4 (10.4–16.6)	52.7 (30.7–93.8)	163.2 (138.0–187.0)	24	49

### Ultrasound examination and sexual maturity rating

Ultrasonographic assessment of BM demonstrated a statistically significant correlation with SMR, regardless of the method used Sailly or Kijima, or the investigators #1 or #2, conducting the assessment. Spearman’s correlation coefficients (ρ) ranged from 0.541 to 0.650, all with p-values < 0.001, indicating significant and consistent associations, see Table 2.

**Table 2:** Correlations (Spearman’s ρ) and 95% confidence intervals between SMR and two ultrasonographic methods (Sailly and Kijima) for assessing biological maturation in both legs. The data are presented separately for two independent investigators

			Spearman’s ρ	95% Confidence Intervals		Significance (2-tailed)
				Lower	Upper	
Independent Investigator #1	Sailly Method	Left Leg	0.541	0.349	0.689	< 0.001
		Right Leg	0.559	0.372	0.703	< 0.001
	Kijima Method	Left Leg	0.596	0.418	0.730	< 0.001
		Right Leg	0.594	0.416	0.728	< 0.001
Independent Investigator #2	Sailly Method	Left Leg	0.647	0.484	0.766	< 0.001
		Right Leg	0.650	0.489	0.769	< 0.001
	Kijima Method	Left Leg	0.611	0.438	0.740	< 0.001
		Right Leg	0.635	0.469	0.758	< 0.001

For Investigator #1, the Sailly method showed moderate correlations with SMR for both the left leg (ρ = 0.541) and right leg (ρ = 0.559). The Kijima method yielded slightly higher correlations: left leg (ρ = 0.596) and right leg (ρ = 0.594).

For Investigator #2, the Sailly method produced correlations of ρ = 0.647 (left) and ρ = 0.650 (right). The Kijima method produced correlations of ρ = 0.611 (left) and ρ = 0.635 (right), as shown in Table 2.

### Inter- and intra-rater reliability

**Fig. 3:**
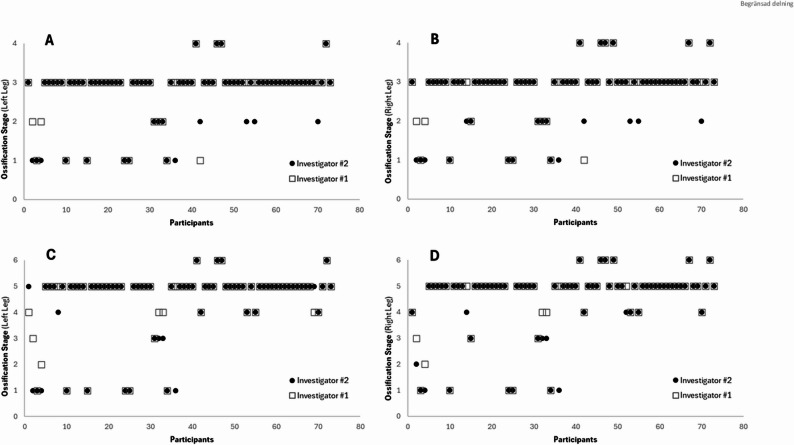
Evaluation of ossification stages by both investigators for all participants (*N* = 73) in both legs, using the Sailly method (Figures **A **and **B**) and the Kijima method (Figures **C **and **D**)

A high degree of inter-rater reliability was observed between the two investigators’ measurements using both the Sailly and Kijima methods. Specifically, the intraclass correlation coefficients indicated substantial agreement: for the Sailly method, Kappa values were 0.767 (left leg) and 0.761 (right leg), both with *p* < 0.001; for the Kijima method, Kappa values were 0.767 (left leg) and 0.814 (right leg), also with *p* < 0.001 (Fig. 3).

A significant intraclass correlation (p<0.001) was identified when inverstigator#1 conducted a reevaluation of 50 randomly selected ultrasonography images (Table 3). Further a significant correlation was observed between the left and right legs, independent of the ultrasonographic method used to assess maturation. Specifically, using the Sailly method, the correlation coefficient was ρ = 0.949 (95% CI: 0.919–0.968, *p* < 0.001), while the Kijima method yielded a correlation of ρ = 0.943 (95% CI: 0.909–0.964, *p* < 0.001).

**Table 3:** Intraclass correlations for both legs and both ultrasonographic methods (Sailly and Kijima)

		Weighted Kappa	95% Confidence Intervals		Significance (2-tailed)
			Lower	Upper	
Sailly method	Left Leg	0.879	0.709	1.049	< 0.001
	Right Leg	1.000	1.000	1.000	< 0.001
Kijima method	Left Leg	0.933	0.835	1.031	< 0.001
	Right Leg	1.000	1.000	1.000	< 0.001

## Discussion

### Validity and reliability of the method

The present study evaluated biological maturity (BM) using two complementary methods: sexual maturity ratings (SMR) conducted by a pediatric endocrinologist and ultrasonographic assessments performed by a physiotherapist. The findings indicate that ultrasonography is a valid and non-inferior alternative to conventional clinical approaches for assessing BM in healthy, physically active youth.

Analyses of inter-rater and intra-rater reliability further support the robustness of ultrasonography. These results align with those of Lv and Zhang [23], who reported exceptionally high inter-rater agreement (ICC = 0.993), as well as with evidence synthesized in a recent scoping review demonstrating that ultrasound-based bone maturity assessments generally achieve high to excellent reliability [24]. Together, these findings suggest that ultrasonographic examination of the tibial tuberosity is both methodologically sound and reproducible when performed by trained professionals.

Ultrasonography also offers practical advantages in pediatric settings. Its non-invasive nature, absence of radiation exposure, and minimal discomfort make it well suited for repeated evaluations. Furthermore, the feasibility of having physiotherapists perform these assessments enhances accessibility and scalability, particularly in environments where specialist resources are limited.

### Methodological strength and practicality

As noted by Rüeger et al. [24], ultrasonographic assessment of the tibial tuberosity enables visualization of maturation stages when conducted by properly trained personnel. The computerized system that aggregates multiple measurements and compares them with normative data [14, 15] adds structure and objectivity to the evaluation process. Although the current findings are promising, additional research is needed to refine reproducibility and to better understand how potential confounding factors might influence automated scoring.

Recent work further supports the utility of ultrasonography as a maturity-assessment tool. Utczas et al. [25], for example, demonstrated substantial concordance between ultrasonography and radiographic bone age, strengthening the case for its use in situations where radiation exposure is undesirable or imaging resources are limited.

### Symmetry and efficiency

The present study identified a substantial correlation between BM assessments of the right and left legs, reinforcing the principle of bilateral skeletal symmetry. This is consistent with findings by Sheng et al. [26] showing comparable bone age estimates between left and right hands.

These results suggest that evaluating a single leg may be sufficient for estimating tibial tuberosity maturation. Although the study does not recommend favoring one side over the other, the potential to reduce assessment time without compromising accuracy represents a meaningful practical advantage, particularly for large-scale or field-based screenings.

### Perspectives and practical applications

Interest in assessing skeletal maturity continues to grow within sports science and pediatric medicine due to strong associations between maturity status, training load, and injury risk [27, 28]. During puberty, discrepancies between chronological and biological age may exceed two years, particularly among girls aged 10–15 and boys aged 12–17, highlighting the limitations of age-based classification systems commonly used in youth sports [29].

Parfitt et al. [30] reported that periods of rapid pubertal growth correspond with temporary reductions in skeletal calcium content, especially in metaphyseal regions. When combined with inappropriate training loads or misclassification in age-grouped competition, these physiological vulnerabilities increase the likelihood of overuse injuries. Rather than discouraging participation, these findings underscore the need to tailor training demands to an adolescent’s biological maturity.

Ultrasound-based assessments offer a practical, field-appropriate tool for estimating BM and can facilitate more individualized training and injury-prevention strategies [30–32]. The present findings suggest that ultrasonography of the tibial tuberosity may help clinicians, coaches, and parents make informed decisions about training progression, recovery, and competition readiness.

While ultrasonography showed substantial agreement with SMR, it was not compared with radiographic bone age, which is the established gold standard. Consequently, ultrasonography should be regarded as a complementary tool rather than a replacement for radiographic methods. Although MRI-based techniques are also capable of assessing skeletal maturation, their cost, availability, and longer acquisition times limit their practicality. This study therefore contributes valuable evidence regarding how established ultrasound protocols relate to SMR-defined maturity in physically active youth.

### Comparison of ultrasound protocols

Clear distinctions emerged between the Sailly and Kijima ultrasound protocols with respect to correlation and reliability. These differences appear to reflect methodological characteristics inherent to each protocol. The Sailly method requires identification of a continuous fascial interface and incorporates dynamic scanning of the iliopsoas–gluteus minimus region, potentially making it more sensitive to subtle maturational differences. This may explain why Investigator #2, who had greater experience with dynamic musculoskeletal ultrasound, achieved higher correlations using the Sailly protocol.

In contrast, the Kijima method utilizes fixed osseous landmarks and a standardized scanning sequence, attributes that likely diminish operator-dependent variability. Such methodological consistency may account for the elevated inter-rater reliability observed in our study and is consistent with previous findings indicating enhanced reproducibility in landmark-based ultrasound protocols [33, 34]. Taken together, these findings suggest that while the Sailly protocol may detect more subtle variations when used by experienced operators, the Kijima method offers greater consistency across examiners, making it advantageous for multicenter or clinician diverse settings.

## Limitations

This study has several limitations. The sample size was relatively small and weighted toward boys, which may restrict the generalizability of the findings across genders. The predominantly Caucasian composition of the sample also raises concerns regarding potential ethnic bias in interpreting BM patterns.

Additionally, the cohort was drawn from a region in northern Sweden characterized by prolonged periods of limited sunlight exposure, which may affect bone maturation. As such, care should be taken when applying these findings to populations in other geographical or environmental contexts [35].

The ultrasonographic assessments were conducted by a physiotherapist with substantial experience in musculoskeletal imaging. Although this ensured high quality data collection, it may limit the generalizability of the findings, as the method has not yet been widely standardized or implemented across varied clinical or athletic environments. Additionally, conditions like Osgood-Schlatter, which is common in young athletes, may increase the risk of inaccurate skeletal maturity assessment via ultrasound.

Finally, while SMR served as the reference standard due to ethical and logistical constraints, radiographic bone age was not included. Although SMR is commonly used and demonstrates acceptable agreement with radiographic methods, it is not equivalent to the imaging gold standard.

## Conclusion

This study demonstrates that ultrasonographic assessment of the tibial tuberosity is a valid, reliable, and practical method for estimating biological maturity in physically active youth. Its non-invasive and radiation-free nature, paired with its portability and cost-effectiveness, makes it well suited for pediatric and sports settings where frequent or large scale assessments may be needed.

By emphasizing biological rather than chronological age, ultrasonography can support individualized training decisions and help reduce the risk of growth related injuries. Future research should prioritize standardization of assessment protocols, validation across more diverse populations, and exploration of the long-term outcomes associated with maturity informed training strategies.

## Data Availability

The datasets generated during and analyzed during the current study are available from the corresponding author upon reasonable request.
